# Mr. Snow's Case of Fractured Skull

**Published:** 1805-04-01

**Authors:** B. G. Snow

**Affiliations:** Farnham, Surry


### To the Editors of the Medical and Physical Journal.

Gentlemen,

I HAVE taken the liberty to transmit you a Case of Fractured Skull, which strikes me as somewhat singular, from so great an extent of injury not requiring the operation of the trepan, which if, in your judgments, you may deem worthy of a place in your monthly publication, I will thank you to insert it. I have not the vanity to suppose, that to the greater part of your readers it can afford the least information; but as I feel myself that I have derived considerable instruction from the case, I conceive it probable that some of the younger branches of the profession may be led, under similar circumstances, to withhold the use of the instruments, and spare their patients a great deal of pain and a tedious recovery. The case is as follows: A soldier, without the least provocation, struck his comrade who was asleep, and lying upon a form rather in liquor, with a flat piece of iron, about two feet and a half in length, and about six pounds weight, upon the parietal bone, two inches and a half in a perpendicular direction above the extreme edge of the external ear; the scalp was divided nearly three inches longitudinally, and the fracture continued the whole length of the wound, together with the depression of the upper edge of the bone, so that the inferior edge could be very distinctly felt, as well as a small piece of the upper table of the skull, loosely detached, but adhering firmly to the scalp. The blow had divided the continuation of the temporal artery; and as it was some length of time before his situation was discovered, when I went down to him, I found he had lost full two pints of blood. After taking up the artery, and finding the man perfectly sensible, I determined not to use the trephine until the symptoms warranted it; and closing the wound by two stitches, left him for the night. In the morning (being December 4th) I was much pleased to see him entirely free from every appearance of coma, delirium, or even fever; I gave him a saline purge, which operated three or four times copiously. In the evening I repeated my visit; I thought his pulse a little too full, although he did not complain of any pain. I took from his arm sixteen ounces of blood, and he expressed himself "lighter" for its loss. I kept him on very low

 diet, diet, and continued seeing him three or four times during the day; no alarming symptoms occurred. On the fifth day from the accident his pulse felt stronger than I wished it, and I was very particular in my inquiries respecting his feelings; but he assured me he felt as well as usual; however, I considered it better, if possible, to prevent the approach of inflammation, and I bled him to sixteen ounces, as before, and no return of fulness existed afterwards. During the whole time of his illness, his appetite continued very good, and his bowels regular; and as no fever or any other bad symptom was induced, I conceived medicines to be useless. On the 21st of December, I reported him convalescent. The wound was healed in part by the second intention, and in part by granulations, and had every favourable appearance throughout; had I been attentive in bringing the edges together the first evening, I have no doubt but the wound would have healed by the first intention; but it was very late, and I thought the operation was inevitable, which made me neglect it. The patient is now perfectly recovered, and except a little debility from the loss of blood, low diet, &c. &c. is as well as if no accident had happened to him. Had I performed the operation immediately, which I think the nature and extent of the injury fully warranted, I certainly might have produced a greater danger, and probably bad consequences, which the event of the case has clearly proved did not exist in any material degree without it.

I am, &c.

*Farnham, Surry, Jan. 2, 1804.*

B. G. SNOW.

